# Imaging and detecting intercellular tensile forces in spheroids and embryoid bodies using lipid-modified DNA probes

**DOI:** 10.3389/fcell.2023.1220079

**Published:** 2023-10-18

**Authors:** Qian Tian, Feiyu Yang, Han Jiang, Priyanka Bhattacharyya, Tianfa Xie, Ahsan Ausaf Ali, Yubing Sun, Mingxu You

**Affiliations:** ^1^ Department of Chemistry, University of Massachusetts Amherst, Amherst, MA, United States; ^2^ Department of Mechanical and Industrial Engineering, University of Massachusetts Amherst, Amherst, MA, United States; ^3^ Molecular and Cellular Biology Program, University of Massachusetts Amherst, Amherst, MA, United States

**Keywords:** cell-cell junction, DNA probes, fluorescence imaging, mechanotransduction, tensile forces, 3D cell model

## Abstract

Cells continuously experience and respond to different physical forces that are used to regulate their physiology and functions. Our ability to measure these mechanical cues is essential for understanding the bases of various mechanosensing and mechanotransduction processes. While multiple strategies have been developed to study mechanical forces within two-dimensional (2D) cell culture monolayers, the force measurement at cell-cell junctions in real three-dimensional (3D) cell models is still pretty rare. Considering that in real biological systems, cells are exposed to forces from 3D directions, measuring these molecular forces in their native environment is thus highly critical for the better understanding of different development and disease processes. We have recently developed a type of DNA-based molecular probe for measuring intercellular tensile forces in 2D cell models. Herein, we will report the further development and first-time usage of these molecular tension probes to visualize and detect mechanical forces within 3D spheroids and embryoid bodies (EBs). These probes can spontaneously anchor onto live cell membranes via the attached lipid moieties. By varying the concentrations of these DNA probes and their incubation time, we have first characterized the kinetics and efficiency of probe penetration and loading onto tumor spheroids and stem cell EBs of different sizes. After optimization, we have further imaged and measured E-cadherin-mediated forces in these 3D spheroids and EBs for the first time. Our results indicated that these DNA-based molecular tension probes can be used to study the spatiotemporal distributions of target mechanotransduction processes. These powerful imaging tools may be potentially applied to fill the gap between ongoing research of biomechanics in 2D systems and that in real 3D cell complexes.

## 1 Introduction

Cells are physically coupled with each other and constantly transmit mechanical signals into downstream biochemical signals and pathways. Mechanical force is an important stimulus that can regulate various cellular processes, such as cell adhesion, proliferation, migration, and tissue development and homeostasis (Yonemura et al., 2010; Provenzano and Keely, 2011; Tambe et al., 2011; Tseng et al., 2012; Heisenberg and Bellaiche, 2013; Streichan et al., 2014; Pandya et al., 2017; Kale et al., 2018; Miroshnikova et al., 2018). Our understanding of the cellular functions of mechanical forces has been dramatically advanced during the past decades, especially due to the development of powerful tools and approaches to *in situ* measure and regulate different cellular mechanosensing and mechanotransduction procedures (Roca-Cusachs et al., 2017; Vining and Mooney, 2017; Du et al., 2023). However, most of these tools and previous studies have been focused on the forces between cells and extracellular matrices (ECM), either in two-dimensional (2D) or three-dimensional (3D) biological samples. For example, traction force microscopy has been popularly used to characterize cellular tractions by measuring beads displacement in the ECM (Maskarinec et al., 2009; Legant et al., 2010; Franck et al., 2011; Polacheck and Chen, 2016; Steinwachs et al., 2016). In addition, different collagen gels and tissue pillars of defined mechanical properties have been applied to measure tissue deformations under different stimuli (Delvoye et al., 1991; Vandenburgh et al., 2008; Legant et al., 2009).

While in sharp contrast, force measurement and regulation between cells and their neighboring cells remains a technical challenge, especially in real 3D environment. These biomechanical communications between individual cells have been shown to play critical roles in collective cell migration and oriented division, cancer cell invasion, wound healing, and various developmental and homeostatic processes (Omelchenko et al., 2003; Schultz et al., 2011; Koch et al., 2012; Heisenberg and Bellaiche, 2013; Pandya et al., 2017; Kale et al., 2018; Li and Wang, 2020), etc. To measure tensile forces at cell-cell junctions, we have recently developed a type of DNA hairpin-based molecular tension probes (Zhao et al., 2017). These DNA nanostructures can sense piconewton-scale mechanical forces and have emerged as powerful tools to measure molecular-level mechanical forces at the interface between cells and ECM (Wang and Ha, 2013; Zhang et al., 2014; Tian et al., 2022). By anchoring these DNA tension probes with a lipid moiety, our lab has demonstrated that these lipid-DNA conjugates can be spontaneously modified onto live cell membranes and used for imaging and quantifying intercellular molecular tensions (Zhao et al., 2017; Zhao et al., 2020a; Keshri et al., 2021).

These lipid-DNA-based molecular tension probes exhibit several unique features. First of all, these probes function by simply incubating with target cells, *in situ* force signals can then be directly visualized in a few minutes. In addition, these probes are highly modular and function in a near plug-and-play configuration. By conjugating different ligand moieties within the DNA probes, specific intercellular ligand-receptor mechanical interactions can be studied (Zhao et al., 2017). Meanwhile, based on the choice of fluorophore-quencher pairs and DNA sequences and lengths, different membrane ligand-receptor pairs or force ranges can be simultaneously detected at the same cell-cell junctions (Zhao et al., 2020a; Keshri et al., 2021). These modular lipid-DNA tension probes are also compatible with easily accessible fluorescence microscopes, which thus hold great potential for broad usage in regular biological labs.

Currently, one major limitation in applying these lipid-DNA molecular tension probes is that they have only been tested in 2D cell culture monolayers. The potential usage of these lipid-DNA probes for detecting intercellular forces in more physiologically relevant 3D cellular environment has not yet been explored. The force measurement at cell-cell junctions in 3D cell models is also still a technical challenge in general. It is worth mentioning that the lipid-DNA conjugates have been successfully used for tissue engineering and *in vivo* therapies (Bagheri et al., 2019b; Zhao et al., 2020b; Nagata et al., 2021), indicating their potential for penetrating into multicellular 3D systems. In this study, we aim to first test the feasibility of modifying lipid-DNA probes onto individual cell membranes within 3D spheroids and embryoid bodies (EBs), and then to test the possibility of applying these lipid-DNA molecular tension probes for imaging and measuring intercellular tensile forces in these 3D cell models, for the first time.

## 2 Materials and methods

### 2.1 Materials

Unless indicated otherwise, all the chemicals were purchased from Fisher Scientific (Waltham, MA, United States) or MilliporeSigma (Burlington, MA, United States). Chemical reagents were directly used without additional purification. All the DNA oligonucleotides were synthesized and HPLC-purified by W. M. Keck Oligonucleotide Synthesis Facility (Yale University School of Medicine) or Integrated DNA Technology (Coralville, IA, United States). Bio-Spin-6 columns were purchased from Bio-Rad (Hercules, CA, United States). Protein G was purchased from Abcam (Cambridge, United Kingdom). Aggrewell 800 microwell culture plates, 73 µm strainer, anti-adhesion rinsing solution, vitronectin XF, and CellAdhere dilution buffer were purchased from STEMCELL Technologies (Vancouver, Canada). BIOFLOAT 96-well ultra-low attachment U-bottom cell culture plate was purchased from faCellitate (Mannheim, Germany).

### 2.2 Cell culture

MCF-7 cells were cultured in DMEM supplemented with 10% FBS and 1% penicillin-streptomycin at 37°C with 5% CO_2_. Standard cell culture procedures were followed for the passage of MCF-7 cells. H9 human embryonic cells (H9; WA09, WiCell) were cultured in Gibco Essential 8 growth medium with its × 50 supplement on human embryonic stem cell-qualified Geltrex or on vitronectin XF (STEMCELL Technology) coated plates (Thermo Fisher Scientific) at 37°C with 5% CO_2_, and the medium was changed every day.

### 2.3 Generation of MCF-7 tumor spheroids

When MCF-7 cells reached 80%–90% confluency, the cells were detached by adding 0.25% trypsin-EDTA. The cell pellet was then resuspended in growth medium and counted with Luna automatic cell counter. Meanwhile, Aggrewell 800 microwell culture plates were coated with 500 µL anti-adhesion solution in each well and centrifuged at 1,300 × g for 5 min to remove air bubbles. The anti-adhesion solution was washed away by basal DMEM medium for three times. Afterwards, 1 × 10^6^ cells were seeded in each well with 2 mL as the final volume. The cells were then centrifuged down at 120 × g for 3 min to ensure their distribution inside the microwells. After overnight incubation at 37°C with 5% CO_2_, the formed MCF-7 spheroids were gently transferred into a 15 mL centrifuge tube with a 73 µm strainer to remove medium and resuspend in Dulbecco’s phosphate buffered saline (DPBS). 100 μL of resuspended spheroids were then transferred into 96-well plate and incubate with different probes for imaging. For long-term spheroid culture, 1 × 10^6^ cells were seeded with 200 µL as the final volume in each well of BIOFLOAT 96-well ultra-low attachment U-bottom cell culture plate. After 5 days of incubation at 37°C with 5% CO_2_, the formed MCF-7 spheroids were imaged directly in these plates after removing medium and resuspending in DPBS.

### 2.4 Generation of stem cell EBs

When H9 human embryonic stem cells reached 80%–90% confluency, the cells were collected using a cell scraper (BD Biosciences) after being treated with 0.5 mM EDTA dissociation buffer. Then the cells were centrifuged at 200 × g for 5 min (Lin and Chen, 2014). The cell pellet was then resuspended in growth medium and counted with Luna automatic cell counter. Meanwhile, Aggrewell 800 microwell culture plates were coated with 500 µL anti-adhesion solution in each well and centrifuged at 1,300 × g for 5 min to remove air bubbles. The anti-adhesion solution was washed away by basal DMEM medium for three times. Afterwards, 1 × 10^6^ cells were seeded in each well with 2 mL as the final volume. 10 μM of Y-27632 ROCK inhibitor (Cayman Chemical) was also supplemented in the well. The cells were then centrifuged down at 120 × g for 3 min to ensure their distribution inside the microwells. After overnight incubation at 37°C with 5% CO_2_, the formed EBs were gently transferred to a 15 mL centrifuge tube with a 73 µm strainer to remove medium and resuspend in DPBS. 100 μL of resuspended EBs were then transferred into 96-well plate and incubate with different probes for imaging.

### 2.5 Synthesis of protein G-modified DNA

The synthesis of protein G-modified DNA strand was performed following a previously developed protocol (Wang et al., 2016; Zhao et al., 2017). Briefly, 20 µL of 200 µM thiol- and FAM-modified ligand strand was mixed with 7 µL Dulbecco’s PBS, 2 µL TCEP, and 1 µL 0.5 mM EDTA and incubated at room temperature for 1 h. Afterwards, the excess TCEP was removed by a Bio-Spin 6 column (Bio-Rad) and 1.5 µL of freshly prepared 1 mg/mL sulfo-SMCC (dissolved in nuclease free water) was then added for 1 min. Immediately after that, 10 µL of 10 mg/mL protein G was added to stop the reaction. The resulting mixture was incubated at 4°C for overnight. On the next day, the protein G-DNA mixture was purified with Dynabeads (Invitrogen). Here, the protein G-DNA mixture was incubated with 50 µL of Dynabeads for 15 min at 4°C on a roller at 40 RPM speed, and after that, unbounded DNAs were removed with washing buffer and magnetic stand. Then, after four times of washing, 100 µL of His elution buffer was added to the protein G-DNA-immobilized Dynabeads and mixed on the roller for 10 min at 4°C at 40 RPM speed. The eluted supernatant was then collected and purified by the Bio-Spin 6 column. The purified product was lastly concentrated with an Amicon 10 kDa ultracentrifuge filter, and the final concentration was measured by a NanoDrop One spectrophotometer. The purified product was either used immediately or kept at −20°C freezer for relatively longer-term storage.

### 2.6 Synthesis of E-cadherin-modified tension probe

The Cy5-modified hairpin strand was first annealed with the Eclipse-/cholesterol-modified anchor strand by heating at 95°C for 5 min and gradually cooling down to room temperature at a rate of 1.3°C/min. Then, the annealed hairpin and anchor strands were mixed with the protein G-modified ligand strand at 1:1 ratio to reach final concentration of 1 µM. The non-quenched (NQ) and quenched (Q) probes without the protein G modifications were prepared using the same approach. The sequences of these DNA strands and illustrated secondary structures of the probes can be found in the Supplementary Material. After the three DNA strands mixture with protein G modification was incubated at 4°C for overnight, E-cadherin was added to the mixture at 1:1 ratio with 500 nM as the final concentration. The E-cadherin-modified DNA probes are now ready for use after incubation at 4°C for overnight and characterization by 10% native polyacrylamide gel.

### 2.7 Fluorescence imaging and data analysis

The above-synthesized MCF-7 spheroids or H9 EBs were first placed in 96-well glass bottom plate (Cellvis, Mountain View, CA, United States) and immersed in 100 µL of DPBS containing 0.5–2 µM lipid-DNA probes and incubated at room temperature (23°C) for at least 30 min before imaging. For the measurement of E-cadherin-mediated intercellular forces and probe modification efficiency, kinetics, and penetration depth, the fluorescence images were taken using a Nikon TiE stand with A1HD (1,024 × 1,024 pixels) resonant scanning confocal microscope at 488 nm and 640 nm laser channels with ×20 air objective. Z-stacking images were taken with a 3 µm spacing for MCF-7 spheroids and a 5 µm spacing for H9 EBs. All the images were analyzed with Nikon NIS analysis software. Determination of the penetration depth was achieved by first drawing lines from randomly chosen periphery region to the central region of the spheroids or EBs with NIS Element Analysis software. By extension, these lines should be merged at the central region of the spheroids or EBs. To measure the penetration depth, the lines stopped where the fluorescence intensity was lower than the threshold, which is mean cellular background fluorescence plus three standard deviations, µ + 3σ. The background fluorescence signals were measured in five random regions from the non-cell part of the same image. Five lines in each image and five images in the z-stacking images of a spheroid/EB with different spacing (6 µm for spheroids and 10 µm for EBs) were analyzed together to obtain the average penetration depth of each corresponding spheroid or EB. All the chosen imaging regions were at least 12 µm higher than the bottom of the spheroids or EBs based on the Atto488 fluorescence signals.

### 2.8 Immunofluorescence

The MCF-7 cells or spheroids were first seeded in 96-well glass bottom plate and after overnight growth, fixed with 4% paraformaldehyde for 30 min. After three times of washing with DPBS, the fixed cells were blocked for 1 h at room temperature with 1% donkey serum containing 0.1% triton X. The blocking solution was then washed away for three times with DPBS. Afterwards, primary E-cadherin antibody was added at a 1:500 ratio for overnight incubation at 4°C. On the next day, the primary antibody was washed away with DPBS for three times and Atto488-labelled secondary antibody was added at a 1:200 ratio for 1 h at room temperature. The immunofluorescence was then imaged with a Leica DMi8 inverted epifluorescence microscope.

## 3 Results

### 3.1 Loading of lipid-DNA conjugates in MCF-7 spheroids

**FIGURE 1:**
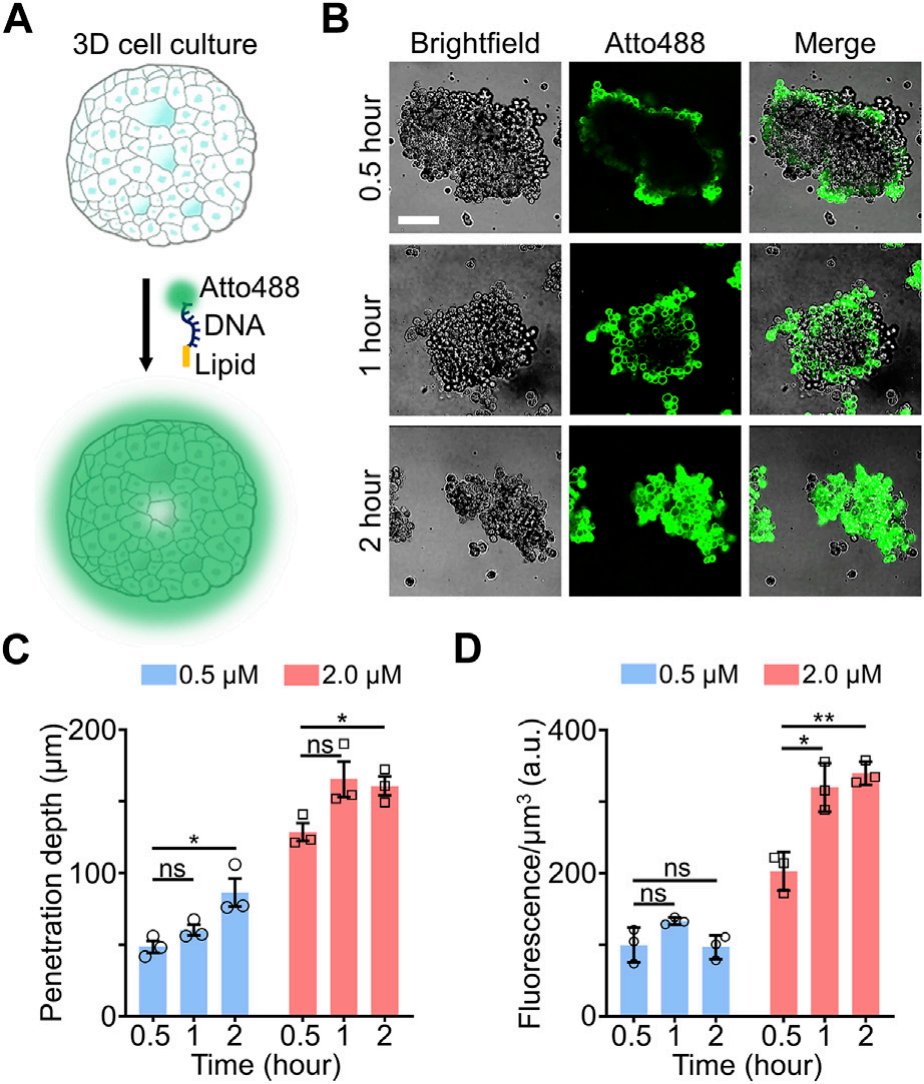
**(A)** Schematic of lipid-DNA modification in 3D cell cultures. **(B)** Representative confocal fluorescence images after incubating 0.5 µM of Atto488-labeled lipid-DNA conjugates with MCF-7 spheroids for 0.5–2.0 h at room temperature. Scale bar, 100 µm. **(C)** The penetration depth of lipid-DNA after incubating 0.5 µM or 2.0 µM of Atto488-labeled lipid-DNA conjugates with MCF-7 spheroids for 0.5–2.0 h at room temperature. **(D)** The average fluorescence intensities of the lipid-DNA modification region after incubating 0.5 µM or 2.0 µM of Atto488-labeled lipid-DNA conjugates with MCF-7 spheroids for 0.5–2.0 h at room temperature. Shown are the mean and standard deviation (SD) values from images taken from at least three spheroids in each case. Two-tailed Student’s t-test: **, *p* < 0.01; *, *p* < 0.05; ns, not significant, *p* > 0.05.

We first wanted to test the feasibility of lipid-mediated DNA anchoring onto live cell membranes within 3D spheroids and EBs (Figure 1A). For this purpose, a 3′-cholesterol-modified 15-nucleotide (nt)-long DNA strand was synthesized, which exhibits no secondary structure and labeled at the 5′-end with an Atto488 dye for imaging (Supplementary Table S1). Breast cancer MCF-7 spheroids were chosen as a 3D cell culture model for studying the loading efficiency and kinetics of these lipid-DNA conjugates. By simply incubating 0.5 µM of lipid-DNA with the MCF-7 spheroids for 30 min, cell membrane fluorescence signals could be clearly observed, but mainly on peripheral MCF-7 cells that were located at the surface of spheroids (Figure 1B). After further incubation for a total of 2 h, more lipid-DNA conjugates anchored into the MCF-7 spheroids, as shown by the ∼40% increased overall fluorescence intensities. In this case, both surface and internal cells of the spheroids were successfully modified with these lipid-DNA conjugates, while most fluorescence signals remained on the membranes of each individual cell (Figure 1B). These results supported that similar to our previous 2D results (Bagheri et al., 2019a; Keshri et al., 2021), cholesterol-modified DNA strands can rapidly insert onto cell membranes within breast cancer spheroids after simple incubation.

To further determine the penetration ability of lipid-DNA in 3D cell cultures, we defined the “penetration depth” here as the average depth at which the fluorescence intensities of lipid-DNA conjugates fall below mean cellular background fluorescence plus three standard deviations, µ + 3σ (Supplementary Figure S1). In the above-mentioned experiments, when we mixed 0.5 µM of lipid-DNA with the small MCF-7 spheroids, the penetration depth was in the range of ∼69–76 µm after a 0.5–2-h incubation (Supplementary Figure S2). For small-sized spheroids, this penetration depth could be deep enough for modifying cells in most regions of the 3D culture. While for larger spheroids (Supplementary Table S2), cells in the central regions may not be reached. Indeed, for MCF-7 spheroids that are larger than 250 μm × 250 μm × 100 µm along the X-Y-Z axis, even after a 2-h incubation, the penetration depth of lipid-DNA was revealed to be at ∼86 ± 24 µm (Figure 1C), with the center part of these large spheroids remained unmodified.

We wondered if the penetration depth of lipid-DNA can be enhanced by simply increasing the concentrations of these lipid-DNA conjugates. To test this, 2 µM of lipid-DNA was incubated with large-sized MCF-7 spheroids (>250 μm × 250 μm × 100 μm, X-Y-Z) for 0.5–2 h. Indeed, more MCF-7 cells within the center regions of these spheroids were successfully labeled as compared to that of 0.5 µM lipid-DNA (Supplementary Figure S3). The penetration depth reached ∼160 µm after 1 h of incubation, which was not further increased afterwards (Figure 1C). To further estimate the amount of lipid-DNA that was inserted in these large-sized spheroids, we also quantified the average fluorescence intensities of the lipid-DNA modification region, i.e., within the penetration depth. As shown in Figure 1D, ∼1.4–2.5 folds higher fluorescence signals were shown after adding 2 µM of lipid-DNA for 1–2 h than that of 0.5 µM during the same period of incubation. These data indicated that both the penetration depth and loading density of lipid-DNA can be directly tuned by changing the initial concentration of these conjugates.

We also tested the loading of these lipid-DNA conjugates in more mature MCF-7 tumor spheroids. For this purpose, MCF-7 cells were seeded in 96-well ultra-low attachment U-bottom cell culture plate and cultured for 5 days. To further maintain the integrity of these obtained MCF-7 spheroids, 2 µM of lipid-DNA were directly added into these U-bottom plate for 0.5–2 h and then *in situ* imaged. As shown in Supplementary Figure S4, the observed penetration depth was increased from ∼85 µm after 0.5 h of incubation to that of ∼150 μm at 2 h, while the average fluorescence intensities within the lipid-DNA modification region were similar during this time period. These results further validated that lipid-DNA can be used for spheroid staining.

### 3.2 Loading of lipid-DNA in human embryonic stem cell EBs

Our next goal was to test if these lipid-DNA conjugates could also be inserted onto the cell membranes of some more sophisticated 3D cell models, such as human embryonic stem cell (hESC)-derived EBs. Unlike tumor spheroids that lack structural stability and organization, EBs derived from stem cells can self-organize and differentiate into specialized cell types, with strong intercellular and cell-matrix interactions (Yin et al., 2016). These stem cell EBs can thus serve as representative 3D cell models to mimic the tissue microenvironment.

**FIGURE 2:**
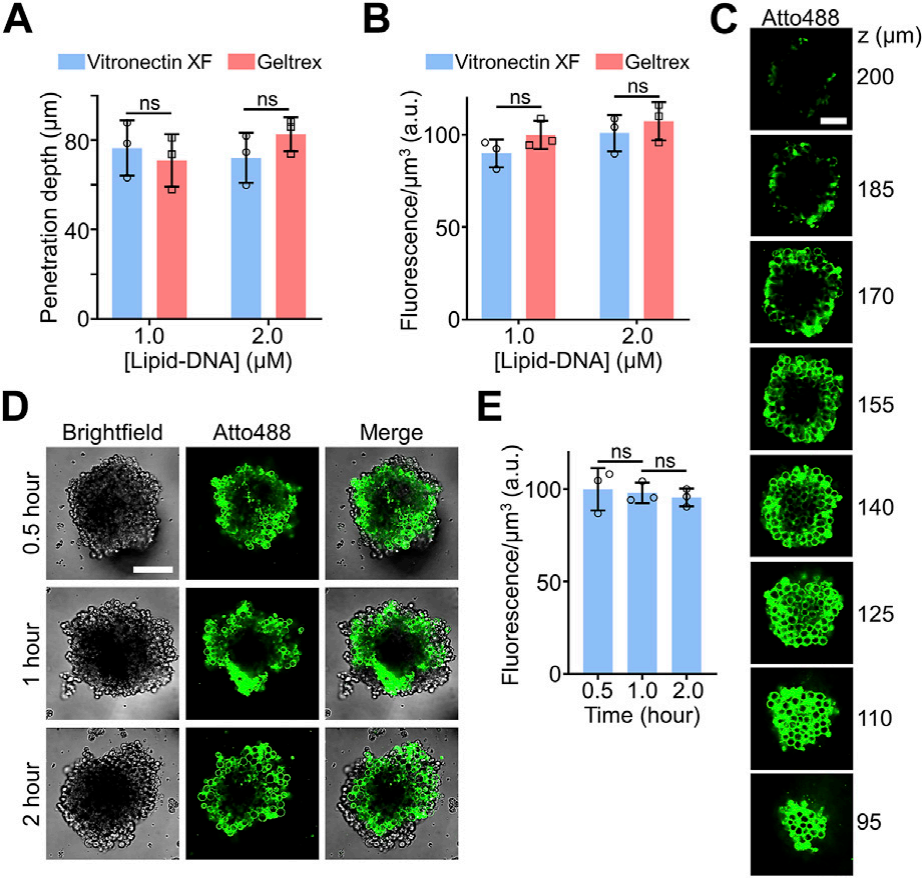
**(A)** The penetration depth of lipid-DNA after incubating 1.0 µM or 2.0 µM of Atto488-labeled lipid-DNA conjugates with H9 hESC embryoid bodies (EBs) for 90 min at room temperature. The Geltrex™ or vitronectin XF™ cell culture matrix was respectively applied to grow these H9 hESC EBs. **(B)** The average fluorescence intensities of the lipid-DNA modification region after incubating 1.0 µM or 2.0 µM of Atto488-labeled lipid-DNA conjugates with Geltrex™- or vitronectin XF™-based H9 hESC EBs for 90 min at room temperature. **(C)** Representative z-stack confocal fluorescence images after incubating 2.0 µM of Atto488-labeled lipid-DNA conjugates with vitronectin XF™-based H9 hESC EBs for 90 min at room temperature. Scale bar, 50 µm. **(D)** Representative confocal fluorescence images after incubating 1.0 µM of Atto488-labeled lipid-DNA conjugates with vitronectin XF™-based H9 hESC EBs for 90 min at room temperature. Scale bar, 100 µm. **(E)** The average fluorescence intensities of the lipid-DNA modification region after incubating 1.0 µM of Atto488-labeled lipid-DNA conjugates with vitronectin XF™-based H9 hESC EBs for 0.5–2.0 h at room temperature. Shown are the mean and standard deviation (SD) values from images taken from at least three EBs in each case. Two-tailed Student’s t-test: ns, not significant, *p* > 0.05.

Here, EBs derived from H9 hESCs were used to study the insertion efficiency and penetration depth of the lipid-DNA conjugates. Two types of cell culture matrices, Geltrex™ and vitronectin XF™, were applied to grow these H9 hESC EBs. After growth, the EBs were further incubated with 1 µM or 2 µM of the above-mentioned 5′-Atto488-3′-cholesterol-modified DNA strands for 90 min to image cellular lipid-DNA modifications. For H9 hESC EBs of comparable size that were generated with either the Geltrex™ or vitronectin XF™ matrix, similar penetration depths and average fluorescence intensities were observed, indicating their similar accessibility to the lipid-DNA conjugates (Figures 2A, B). In addition, 1 μM and 2 µM of lipid-DNA did not exhibit significant difference in their cell modification efficiency, while most of the fluorescence signals were detected on the membranes of individual H9 cells (Supplementary Figure S5).

Compared to the efficiency of labeling the central cells within the MCF-7 spheroids (Figure 1C), after a similar 1–2 h of incubation with 2 µM of lipid-DNA, the penetration depth of the same lipid-DNA conjugate was notably reduced, from ∼160 μm to ∼80 µm (Figure 2A). This result could be correlated with the more organized and compact microenvironments within these H9 hESC-derived EBs. As shown in the z-stack confocal images, under this incubation condition, these lipid-DNA conjugates can still be efficiently modified onto the membranes of most of the cells within the EBs (Figure 2C). While it is worth mentioning that due to the large heights of these synthesized EBs and the limited working distance of high numerical aperture objective, the top region of the EBs (e.g., z > 170 µm) was gradually out of focus, which fluorescence intensities may not represent the real lipid-DNA anchoring efficiencies.

We also studied the kinetics of lipid-DNA modification. After adding 1 µM of lipid-DNA to the H9 hESC EBs that were grown with the vitronectin XF, within 30 min of incubation, cell membrane fluorescence signals could be clearly observed (Figure 2D). Similar average fluorescence intensities within the penetration depth were shown during a total of 2-h incubation time (Figure 2E). All these data together demonstrated that the lipid-DNA conjugates could efficiently penetrate and modify onto the cell membranes within complex 3D cell culture models, such as the hESC EBs.

### 3.3 DNA-based molecular tension probes for measuring intercellular tensile forces in 3D cell cultures

**FIGURE 3:**
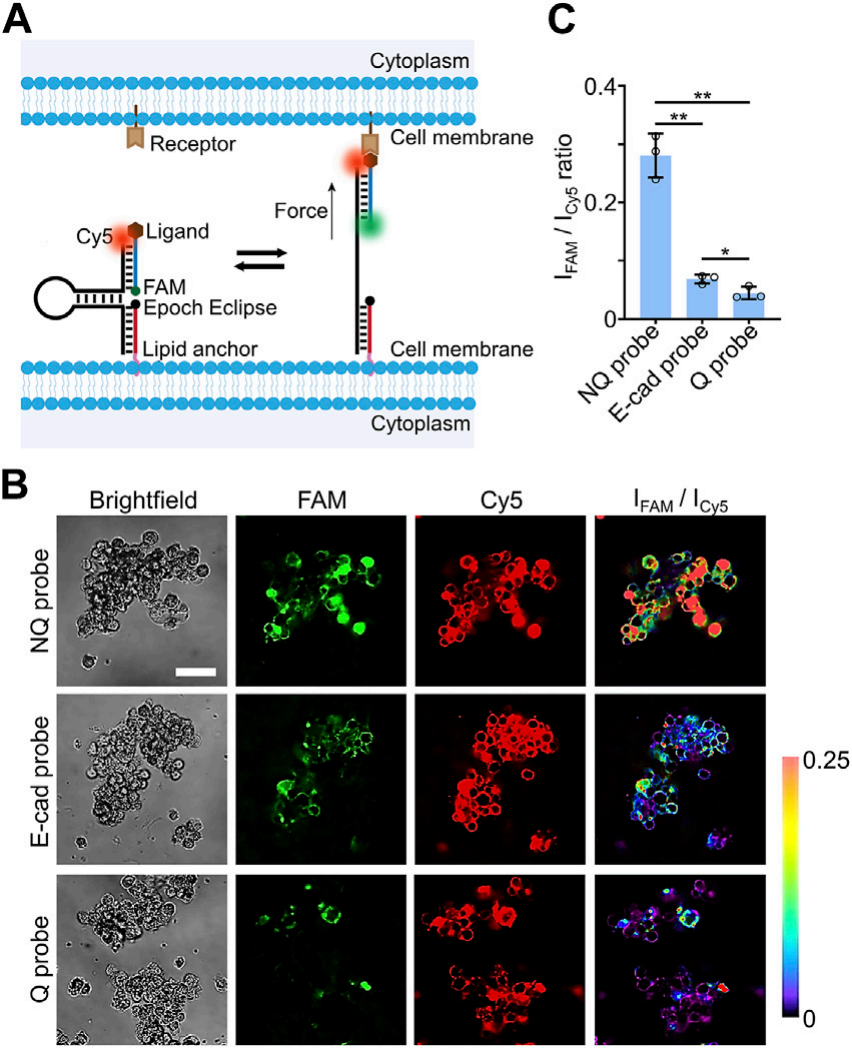
**(A)** Schematic of DNA-based molecular tension probe that consists of three DNA strands, including anchor, ligand, and hairpin strands. **(B)** Representative confocal fluorescence images and pseudo-colored FAM/Cy5 ratiometric images after incubating 0.5 µM of Q probe, NQ probe, or E-cadherin-modified DNA molecular tension probe with MCF-7 spheroids for 30 min at room temperature. Scale bar, 50 µm. **(C)** The average FAM/Cy5 ratiometric fluorescence intensities within MCF-7 spheroids after incubation with 0.5 µM of Q, NQ, and E-cad probe for 30 min at room temperature. Shown are the mean and standard deviation (SD) values from images taken from at least three spheroids in each case. Two-tailed Student’s t-test: **, *p* < 0.01; *, *p* < 0.05.

After characterizing the modification kinetics and efficiencies of the lipid-DNA conjugates on the MCF-7 spheroids and H9 hESC-derived EBs, we attempted to apply these cholesterol-modified DNA strands to engineer fluorescent probes for imaging intercellular molecular tensions within 3D spheroid and EB cell models. As shown in Figure 3A, our DNA-based molecular tension probe consists of three DNA strands: a cholesterol- and Epoch Eclipse quencher-modified anchor strand for the cell membrane insertion, a ligand- and FAM dye-modified strand for targeting and reporting specific intercellular ligand-receptor mechanical interactions, and a Cy5 dye-modified DNA hairpin strand that can hybridize with both the anchor strand and ligand strand to assemble all these DNA oligonucleotides together into a nanodevice.

The Cy5 dye was used as an internal reference here to indicate the loading densities and distributions of these DNA probes. In the absence of tensile forces, the FAM fluorescence signal was quenched by the nearby Epoch Eclipse quencher. While once the ligand experienced forces that are strong enough to unfold the DNA hairpin, a dramatic increase in the FAM fluorescence will be resulted from the separation of the FAM-Epoch Eclipse dye-quencher pair. Therefore, similar to that shown in our previous study in the 2D cell cultures (Zhao et al., 2020a), the FAM-to-Cy5 ratiometric fluorescence signals can be potentially used to image and measure molecular tensile forces at cell-cell junctions.

To test the feasibility of these DNA-based molecular tension probes for measuring intercellular tensile forces within spheroids and EBs, our previously engineered DNA hairpin design with 22% G/C base pairs (Keshri et al., 2021) was applied here to measure E-cadherin-mediated tensions in the 3D cell models. These 22%GC tension probes exhibit a force threshold (F_1/2_) value of ∼4.4 pN, where F_1/2_ represents the force level at which ∼50% of the DNA hairpins can be unfolded. After annealing, the successful self-assembly of the anchor, ligand, and hairpin DNA strands were first validated in 10% native polyacrylamide gel electrophoresis (Supplementary Figures S6A). Meanwhile, a ∼65% quenching efficiency in the FAM channel was observed once the Epoch Eclipse quencher-modified anchor strand was hybridized together with the hairpin/ligand conjugate in the solution (Supplementary Figures S6B), which further validated the proper folding of DNA-based molecular tension probes.

Next, we measured the cellular fluorescence signals of these molecular tension probes (named as the **Q** probe) in both FAM and Cy5 channels. A quencher-free version of the same three-DNA-strand conjugates was used as a control here, which was named as the **NQ** probe (Supplementary Figures S6C). In this test, 0.5 µM of the Q probe and NQ probe was separately incubated with the MCF-7 spheroids for 30 min before imaging. Based on the fluorescence signals of the Cy5 reference dye, most of the cell membranes throughout the whole MCF-7 spheroids were able to be modified with these DNA probes (Figure 3B). Similar to cholesterol-modified 15-nt-long single-stranded DNA, these three-DNA-strand conjugates can also efficiently penetrate and label central cells within the spheroids. After subtracting the background fluorescence, we compared the average FAM/Cy5 fluorescence intensity ratios of the Q and NQ probes within the MCF-7 spheroids. As shown in Figure 3C, a ∼6.3-fold higher ratiometric signal was observed using the NQ probe than that of the Q probe, which demonstrated a high quenching efficiency of the FAM signals in the absence of mechanical forces.

After further conjugating Fc domain-fused E-cadherin with the DNA tension probe via a protein G linker, 0.5 µM of the prepared E-cadherin-modified probes (called **E-cad** probe) were then incubated with the MCF-7 spheroids for 30 min to measure E-cadherin-mediated intercellular tensions. Indeed, the resulted FAM/Cy5 fluorescence ratio of the E-cad probe was significantly higher than that of the Q probe (Figure 3C), while by comparing with the ratiometric signals of the NQ probe, the percentage of unfolded E-cad probes was not high in these MCF-7 spheroids. Our immunofluorescence staining results indicated that this relatively low level of mechanotransduction was likely not due to the low-expression levels of membrane E-cadherins (Supplementary Figure S7). Instead, the magnitude of E-cadherin-mediated intercellular tensions was not high in the MCF-7 spheroids. Interestingly, a heterogeneous force distribution within these spheroids was observed based on the pseudo-colored FAM/Cy5 ratiometric images (Figure 3B). Some studies may be followed in the future to investigate the possible correlations between these intercellular tensions and some localized cell signaling events.

**FIGURE 4:**
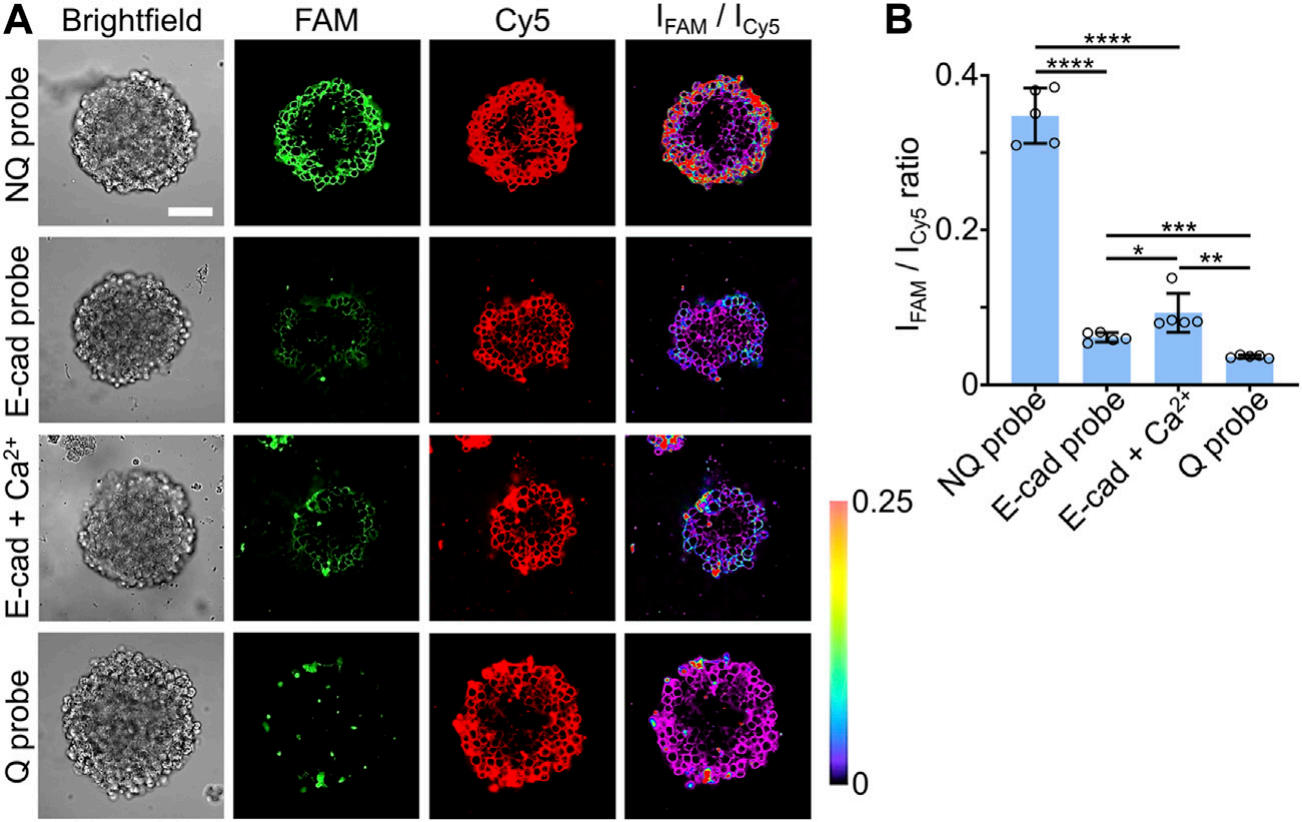
**(A)** Representative confocal fluorescence images and pseudo-colored FAM/Cy5 ratiometric images after incubating 1.0 µM of Q probe, NQ probe, E-cad probe, or E-cad probe and 2 mM of Ca^2+^, with vitronectin XF™-based H9 hESC EBs for 30 min at room temperature. Scale bar, 100 µm. **(B)** The average FAM/Cy5 ratiometric fluorescence intensities within H9 hESC EBs after incubation with 1.0 µM of Q, NQ, E-cad probe, or E-cad probe and 2 mM of Ca^2+^, for 30 min at room temperature. Shown are the mean and standard deviation (SD) values from images taken from at least three EBs in each case. Two-tailed Student’s t-test: ****, *p* < 0.0001; ***, *p* < 0.001; **, *p* < 0.01; *, *p* < 0.05.

We also applied these E-cadherin-modified DNA tension probes to image intercellular tensile forces within the H9 hESC-derived EBs. As shown based on the fluorescence signals of the Cy5 reference (Figure 4A), these Q, NQ, and E-cad molecular probes exhibited similar penetration depths and loading efficiencies as those 15-nt-long lipid-DNA conjugate (Figure 2). After a 30-min incubation of 1 µM DNA probes with the H9 hESC EBs, based on the ratiometric FAM/Cy5 fluorescence signals, E-cadherin-mediated forces could be observed and measured at individual cell-cell junctions in these stem cell EBs (Figure 4).

It is known that calcium ions can be used to promote the E-cadherin interactions at cell-cell junctions (Pokutta et al., 1994; Rothen-Rutishauser et al., 2002). To further validate the function of our DNA probes in measuring intercellular tensions, we added 2 mM of Ca^2+^ ions together with 1 μM E-cad probes to the H9 hESC EBs and incubated for 30 min. As expected, the addition of calcium ions could enhance the E-cadherin-mediated forces in these stem cell EBs, with an enhanced FAM/Cy5 ratiometric fluorescence signals shown on the cell membranes (Figure 4). We also applied these DNA probes to study the effect of blebbistatin, a myosin II inhibitor, on perturbing E-cadherin-mediated forces in these stem cell EBs. After a 10-min treatment with 25 µM of blebbistatin, a decreased FAM/Cy5 ratiometric fluorescence signal was clearly observed (Supplementary Figure S8), validating the contribution of myosin contractibility in the generation of E-cadherin-mediated forces in these EB samples. All these data supported the idea that these lipid-anchored DNA molecular tension probes can indeed be applied to visualize and characterize intercellular tensile forces in different 3D cell models.

Our results also indicated that cells in the periphery regions of the H9 hESC EBs tended to have a higher percentage of tension-induced unfolding of the E-cad probes. In contrast, as the cells were located closer to the centers, lower levels of the FAM/Cy5 ratiometric fluorescence signals will be observed (Figure 4A). These data may suggest that different magnitudes of E-cadherin-mediated forces were distributed in these EB samples. However, for real quantification of the force intensities and unfolding percentage of the molecular tension probes, more careful probe calibration is still needed in the future, similar to that shown in our previous studies (Zhao et al., 2020a; Keshri et al., 2021).

## 4 Discussion

In this study, we demonstrated the function of lipid-DNA molecular tension probes in visualizing and detecting intercellular forces in 3D spheroid and EB cell models. With simple incubation, these probes can spontaneously diffuse and insert onto the membranes of individual cells within these 3D cell cultures. Lipid-DNA conjugates can modify both MCF-7 spheroids and H9 hESC-derived EBs with high modification efficiency and large penetration depth. Notably, the penetration ability of the short 15-nt-long lipid-DNA and the relatively large E-cadherin/protein G-modified DNA tension probe is quite similar to each other, which indicates that the loading efficiency of these lipid-DNA conjugates may be largely determined by the lipid moiety, instead of the molecular weight of the whole conjugate. By changing the type and number of lipid moieties, the penetration depth and modification efficiency of these lipid-DNA probes within the spheroids and EBs could be possibly further improved.

Here, E-cadherin-mediated intercellular tension was investigated as a proof of concept. The same DNA probe design can be modularly and easily modified to study other ligand-receptor pairs of interest following different mechanotransduction pathways. These DNA tension probes can provide spatiotemporal information about intercellular mechanical forces, which could be potentially used for studying the dynamics and functions of mechanotransduction in diverse collective cellular and tissue development processes. It is also possible to further apply these DNA tension probes for measuring multiple magnitudes of forces and multiple molecular targets simultaneously in these 3D cell models (Zhao et al., 2020a; Keshri et al., 2021).

With further testing and optimization, these modular and easy-to-use lipid-DNA molecular tension probes could be possibly used in diverse types of 3D spheroid and EB models, or even real primary tissue samples. We believe these novel tools can be versatilely applied to fill the current gap between 2D biomechanical studies and that in real multilayered 3D cell complexes. In addition to their applications in mechanobiology, by anchoring different DNAs and other ligands onto the cell membranes of spheroids and EBs, these 3D cell model-compatible lipid-DNA conjugates may also be adapted for applications in the field of biosensing, drug delivery, and tissue engineering, etc.

## Data Availability

The original contributions presented in the study are included in the article/Supplementary Material, further inquiries can be directed to the corresponding authors.

## References

[B1] Bagheri Y. Chedid S. Shafiei F. Zhao B. You M. (2019a). A quantitative assessment of the dynamic modification of lipid-DNA probes on live cell membranes. Chem. Sci. 10 (48), 11030–11040. 10.1039/c9sc04251b 32055389 PMC7003967

[B2] Bagheri Y. Shafiei F. Chedid S. Zhao B. You M. (2019b). Lipid-DNA conjugates for cell membrane modification, analysis, and regulation. Supramol. Chem. 31 (8), 532–544. 10.1080/10610278.2019.1632454

[B3] Delvoye P. Wiliquet P. Leveque J. L. Nusgens B. V. Lapiere C. M. (1991). Measurement of mechanical forces generated by skin fibroblasts embedded in a three-dimensional collagen gel. J. Invest. Dermatol. 97 (5), 898–902. 10.1111/1523-1747.ep12491651 1919053

[B4] Du H. Bartleson J. M. Butenko S. Alonso V. Liu W. F. Winer D. A. (2023). Tuning immunity through tissue mechanotransduction. Nat. Rev. Immunol. 23 (3), 174–188. 10.1038/s41577-022-00761-w 35974148 PMC9379893

[B5] Franck C. Maskarinec S. A. Tirrell D. A. Ravichandran G. (2011). Three-dimensional traction force microscopy: a new tool for quantifying cell-matrix interactions. Plos One 6 (3), e17833. 10.1371/journal.pone.0017833 21468318 PMC3066163

[B6] Heisenberg C. P. Bellaiche Y. (2013). Forces in tissue morphogenesis and patterning. Cell 153 (5), 948–962. 10.1016/j.cell.2013.05.008 23706734

[B7] Kale G. R. Yang X. B. Philippe J. M. Mani M. Lenne P. F. Lecuit T. (2018). Distinct contributions of tensile and shear stress on E-cadherin levels during morphogenesis. Nat. Commun. 9, 5021. 10.1038/s41467-018-07448-8 30479400 PMC6258672

[B8] Keshri P. Zhao B. Xie T. F. Bagheri Y. Chambers J. Sun Y. B. (2021). Quantitative and multiplexed fluorescence lifetime imaging of intercellular tensile forces. Angew. Chem. Int. Ed. 60 (28), 15548–15555. 10.1002/anie.202103986 PMC8238903 33961329

[B9] Koch T. M. Munster S. Bonakdar N. Butler J. P. Fabry B. (2012). 3D traction forces in cancer cell invasion. Plos One 7 (3), e33476. 10.1371/journal.pone.0033476 22479403 PMC3316584

[B10] Legant W. R. Miller J. S. Blakely B. L. Cohen D. M. Genin G. M. Chen C. S. (2010). Measurement of mechanical tractions exerted by cells in three-dimensional matrices. Nat. Methods 7 (12), 969–971. 10.1038/Nmeth.1531 21076420 PMC3056435

[B11] Legant W. R. Pathak A. Yang M. T. Deshpande V. S. McMeeking R. M. Chen C. S. (2009). Microfabricated tissue gauges to measure and manipulate forces from 3D microtissues. Proc. Nat. Acad. Sci. U. S. A. 106 (25), 10097–10102. 10.1073/pnas.0900174106 PMC2700905 19541627

[B12] Li X. C. Wang J. L. (2020). Mechanical tumor microenvironment and transduction: cytoskeleton mediates cancer cell invasion and metastasis. Int. J. Biol. Sci. 16 (12), 2014–2028. 10.7150/ijbs.44943 32549750 PMC7294938

[B13] Lin Y. Chen G. (2014). Embryoid body formation from human pluripotent stem cells in chemically defined E8 media. StemBook. 10.3824/stembook.1.98.1 28211653

[B14] Maskarinec S. A. Franck C. Tirrell D. A. Ravichandran G. (2009). Quantifying cellular traction forces in three dimensions. Proc. Nat. Acad. Sci. U. S. A. 106 (52), 22108–22113. 10.1073/pnas.0904565106 PMC2799761 20018765

[B15] Miroshnikova Y. A. Le H. Q. Schneider D. Thalheim T. Rubsam M. Bremicker N. (2018). Adhesion forces and cortical tension couple cell proliferation and differentiation to drive epidermal stratification. Nat. Cell Biol. 20 (1), 69–80. 10.1038/s41556-017-0005-z 29230016

[B16] Nagata T. Dwyer C. A. Yoshida-Tanaka K. Ihara K. Ohyagi M. Kaburagi H. (2021). Cholesterol-functionalized DNA/RNA heteroduplexes cross the blood-brain barrier and knock down genes in the rodent CNS. Nat. Biotechnol. 39 (12), 1529–1536. 10.1038/s41587-021-00972-x 34385691

[B17] Omelchenko T. Vasiliev J. M. Gelfand I. M. Feder H. H. Bonder E. M. (2003). Rho-dependent formation of epithelial "leader" cells during wound healing. Proc. Nat. Acad. Sci. U. S. A. 100 (19), 10788–10793. 10.1073/pnas.1834401100 PMC196881 12960404

[B18] Pandya P. Orgaz J. L. Sanz-Moreno V. (2017). Actomyosin contractility and collective migration: may the force be with you. Curr. Opin. Cell Biol. 48, 87–96. 10.1016/j.ceb.2017.06.006 28715714 PMC6137077

[B19] Pokutta S. Herrenknecht K. Kemler R. Engel J. (1994). Conformational-changes of the recombinant extracellular domain of E-cadherin upon calcium-binding. Eur. J. Biochem. 223 (3), 1019–1026. 10.1111/j.1432-1033.1994.tb19080.x 8055942

[B20] Polacheck W. J. Chen C. S. (2016). Measuring cell-generated forces: a guide to the available tools. Nat. Methods 13 (5), 415–423. 10.1038/Nmeth.3834 27123817 PMC5474291

[B21] Provenzano P. P. Keely P. J. (2011). Mechanical signaling through the cytoskeleton regulates cell proliferation by coordinated focal adhesion and Rho GTPase signaling. J. Cell Sci. 124 (8), 1195–1205. 10.1242/jcs.067009 21444750 PMC3065381

[B22] Roca-Cusachs P. Conte V. Trepat X. (2017). Quantifying forces in cell biology. Nat. Cell Biol. 19 (7), 742–751. 10.1038/ncb3564 28628082

[B23] Rothen-Rutishauser B. Riesen F. K. Braun A. Gunthert M. Wunderli-Allenspach H. (2002). Dynamics of tight and adherens junctions under EGTA treatment. J. Membr. Biol. 188 (2), 151–162. 10.1007/s00232-001-0182-2 12172640

[B24] Schultz G. S. Davidson J. M. Kirsner R. S. Bornstein P. Herman I. M. (2011). Dynamic reciprocity in the wound microenvironment. Wound Repair Regen. 19 (2), 134–148. 10.1111/j.1524-475X.2011.00673.x 21362080 PMC3051353

[B25] Steinwachs J. Metzner C. Skodzek K. Lang N. Thievessen I. Mark C. (2016). Three-dimensional force microscopy of cells in biopolymer networks. Nat. Methods 13 (2), 171–176. 10.1038/Nmeth.3685 26641311

[B26] Streichan S. J. Hoerner C. R. Schneidt T. Holzer D. Hufnagel L. (2014). Spatial constraints control cell proliferation in tissues. Proc. Nat. Acad. Sci. U. S. A. 111 (15), 5586–5591. 10.1073/pnas.1323016111 PMC3992650 24706777

[B27] Tambe D. T. Hardin C. C. Angelini T. E. Rajendran K. Park C. Y. Serra-Picamal X. (2011). Collective cell guidance by cooperative intercellular forces. Nat. Mat. 10 (6), 469–475. 10.1038/Nmat3025 PMC3135682 21602808

[B28] Tian Q. Keshri P. You M. X. (2022). Recent developments in DNA-based mechanical nanodevices. Chem. Commun. 58 (30), 4700–4710. 10.1039/d2cc00302c PMC9007884 35322846

[B29] Tseng Q. Z. Duchemin-Pelletier E. Deshiere A. Balland M. Guillou H. Filhol O. (2012). Spatial organization of the extracellular matrix regulates cell-cell junction positioning. Proc. Nat. Acad. Sci. U. S. A. 109 (5), 1506–1511. 10.1073/pnas.1106377109 PMC3277177 22307605

[B30] Vandenburgh H. Shansky J. Benesch-Lee F. Barbata V. Reid J. Thorrez L. (2008). Drug-screening platform based on the contractility of tissue-engineered muscle. Muscle Nerve 37 (4), 438–447. 10.1002/mus.20931 18236465

[B31] Vining K. H. Mooney D. J. (2017). Mechanical forces direct stem cell behaviour in development and regeneration. Nat. Rev. Mol. Cell Biol. 18 (12), 728–742. 10.1038/nrm.2017.108 29115301 PMC5803560

[B32] Wang X. F. Ha T. (2013). Defining single molecular forces required to activate integrin and Notch signaling. Science 340 (6135), 991–994. 10.1126/science.1231041 23704575 PMC3710701

[B33] Wang X. F. Rahil Z. Li I. T. S. Chowdhury F. Leckband D. E. Chemla Y. R. (2016). Constructing modular and universal single molecule tension sensor using protein G to study mechano-sensitive receptors. Sci. Rep. 6, 21584. 10.1038/srep21584 26875524 PMC4753514

[B34] Yin X. L. Mead B. E. Safaee H. Langer R. Karp J. M. Levy O. (2016). Engineering stem cell organoids. Cell Stem Cell 18 (1), 25–38. 10.1016/j.stem.2015.12.005 26748754 PMC4728053

[B35] Yonemura S. Wada Y. Watanabe T. Nagafuchi A. Shibata M. (2010). alpha-Catenin as a tension transducer that induces adherens junction development. Nat. Cell Biol. 12 (6), 533–542. 10.1038/ncb2055 20453849

[B36] Zhang Y. Ge C. H. Zhu C. Salaita K. (2014). DNA-based digital tension probes reveal integrin forces during early cell adhesion. Nat. Commun. 5, 5167. 10.1038/ncomms6167 25342432 PMC4209443

[B37] Zhao B. Li N. W. Xie T. F. Bagheri Y. Liang C. W. Keshri P. (2020a). Quantifying tensile forces at cell-cell junctions with a DNA-based fluorescent probe. Chem. Sci. 11 (32), 8558–8566. 10.1039/d0sc01455a 34123115 PMC8163409

[B38] Zhao B. O'Brien C. Mudiyanselage A. P. K. K. K. Li N. W. Bagheri Y. Wu R. (2017). Visualizing intercellular tensile forces by DNA-based membrane molecular probes. J. Am. Chem. Soc. 139 (50), 18182–18185. 10.1021/jacs.7b11176 29211468

[B39] Zhao B. Tian Q. Bagheri Y. You M. X. (2020b). Lipid-oligonucleotide conjugates for simple and efficient cell membrane engineering and bioanalysis. Curr. Opin. Biomed. Eng. 13, 76–83. 10.1016/j.cobme.2019.12.006 32642625 PMC7343234

