# Supplementary material for: Imaging and detecting intercellular tensile forces in spheroids and embryoid bodies using lipid-modified DNA probes
Source: Front Cell Dev Biol. 2023 Oct 18;11:1220079. doi: 10.3389/fcell.2023.1220079 (PMC10619156; doi:10.3389/fcell.2023.1220079)
Supplement: Supplementary file 2 [file DataSheet1.DOCX]

Supplementary Material

Imaging and detecting intercellular tensile forces in spheroids and embryoid bodies using lipid-modified DNA probes

Qian Tian, Feiyu Yang, Han Jiang, Priyanka Bhattacharyya, Tianfa Xie, Ahsan Ausaf Ali, Yubing Sun*, Mingxu You*

*** Correspondence:** Yubing Sun: [ybsun@umass.edu](mailto:ybsun@umass.edu) Mingxu You: [mingxuyou@umass.edu](mailto:mingxuyou@umass.edu)

# Supplementary Tables

| **Table S1. Sequences of DNAs used in the study** | |
| --- | --- |
| **Name** | **DNA sequences (5’–3’)** |
| Hairpin strand | CCC GTG AAA TAC CGC ACA GAT GCG TTT GTA TAA ATG TTT TTT TCA TTT ATA CTT TAA GAG CGC CAC GTA GCC CAG C-Cy5 |
| Ligand strand | Thiol-TTT GCT GGG CTA CGT GGC GCT CTT-FAM |
| Anchor strand | Epoch Eclipse-CGC ATC TGT GCG GTA TTT CAC CCC-Cholesterol |
| Non-quenched (NQ) anchor strand | CGC ATC TGT GCG GTA TTT CAC CCC-Cholesterol |
| Lipid-DNA-Atto488 | Atto488-TCT ACA TAC AAC TAC-Cholesterol |

| **Table S2. Dimensions of the MCF-7 spheroids and embryoid bodies**, which were used for the analysis of lipid-DNA penetration depth. These X- and Y-axis diameter values were measured along the estimated “equatorial plane” of each MCF-7 spheroid and embryoid body. | | | | | |
| --- | --- | --- | --- | --- | --- |
| **Name** | **Incubation time (h)** | **X (µm)** | **Y (µm)** | **Z (µm)** | **Figures** |
| 0.5 µM lipid-DNA on small MCF-7 spheroids | 0.5 | 237 | 166 | 95 | Figure S2A |
|  |  | 225 | 119 | 85 |  |
|  |  | 221 | 186 | 75 |  |
|  | 1 | 217 | 174 | 85 |  |
|  |  | 243 | 217 | 80 |  |
|  |  | 182 | 170 | 80 |  |
|  | 2 | 235 | 189 | 80 |  |
|  |  | 195 | 174 | 85 |  |
|  |  | 242 | 197 | 70 |  |
| 0.5 µM lipid-DNA on large MCF-7 spheroids | 0.5 | 310 | 271 | 100 | Figure 1C |
|  |  | 454 | 382 | 110 |  |
|  |  | 402 | 313 | 115 |  |
|  | 1 | 498 | 343 | 100 |  |
|  |  | 489 | 328 | 115 |  |
|  |  | 400 | 346 | 125 |  |
|  | 2 | 497 | 356 | 100 |  |
|  |  | 376 | 265 | 100 |  |
|  |  | 387 | 268 | 105 |  |
| 2 µM lipid-DNA on large MCF-7 spheroids | 0.5 | 436 | 273 | 100 |  |
|  |  | 404 | 388 | 110 |  |
|  |  | 353 | 292 | 110 |  |
|  | 1 | 448 | 383 | 110 |  |
|  |  | 387 | 351 | 105 |  |
|  |  | 382 | 375 | 110 |  |
|  | 2 | 399 | 258 | 105 |  |
|  |  | 605 | 399 | 130 |  |
|  |  | 397 | 337 | 110 |  |
| 2 µM lipid-DNA on 5-day MCF-7 spheroids | 2 | 562 | 399 | 140 | Figure S4C |
|  |  | 473 | 349 | 130 |  |
|  |  | 552 | 397 | 110 |  |
| 1 µM lipid-DNA on Geltrex EBs | 1.5 | 334 | 326 | 140 | Figure 2A |
|  |  | 334 | 329 | 175 |  |
|  |  | 310 | 281 | 135 |  |
| 2 µM lipid-DNA on Geltrex EBs | 1.5 | 331 | 329 | 155 |  |
|  |  | 325 | 308 | 150 |  |
|  |  | 324 | 310 | 155 |  |
| 1 µM lipid-DNA on VNXF EBs | 1.5 | 328 | 326 | 145 |  |
|  |  | 344 | 328 | 140 |  |
|  |  | 341 | 330 | 145 |  |
| 2 µM lipid-DNA on VNXF EBs | 1.5 | 339 | 299 | 160 |  |
|  |  | 319 | 291 | 150 |  |
|  |  | 328 | 314 | 130 |  |

**2 Supplementary Figures**


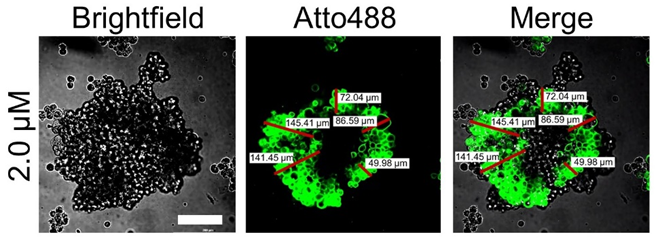


**Supplementary Figure 1.** Representative confocal fluorescence images after incubating 2.0 µM of Atto488-labeled lipid-DNA conjugates with MCF-7 spheroids for 30 minutes at room temperature. Scale bar, 100 µm. To measure the penetration depth, a line (red) was drawn from the periphery to the central region of the spheroids/embryoid bodies (EBs). The lines stopped where the fluorescence intensity was lower than the threshold, which is mean cellular background fluorescence plus three standard deviations, µ + 3σ. The background fluorescence signals were measured in five random regions from the non-cell part of the same image. Five lines in each image were randomly chosen and five images in the z-stacking images of a spheroid/EB with different spacing (6 µm for spheroids and 10 µm for EBs) were analyzed to obtain the average penetration depth.


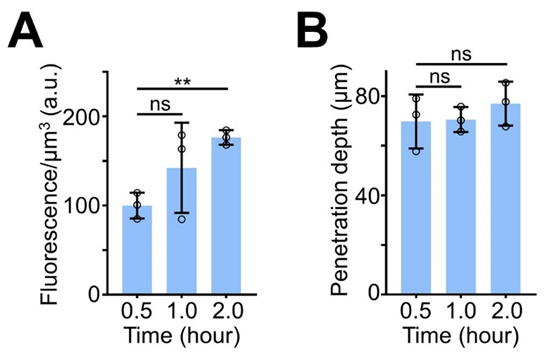


**Supplementary Figure 2. (A)** The average fluorescence intensities of the lipid-DNA modification region after incubating 0.5 µM of Atto488-labeled lipid-DNA conjugates with small-sized MCF-7 spheroids for 0.5–2.0 hours at room temperature. **(B)** The penetration depth of lipid-DNA after incubating 0.5 µM of Atto488-labeled lipid-DNA conjugates with small-sized MCF-7 spheroids for 0.5–2.0 hours at room temperature. Shown are the mean and standard deviation (SD) values from images taken from at least three spheroids in each case. Two-tailed student’s t-test: **, p<0.01; ns, not significant, p>0.05.

**
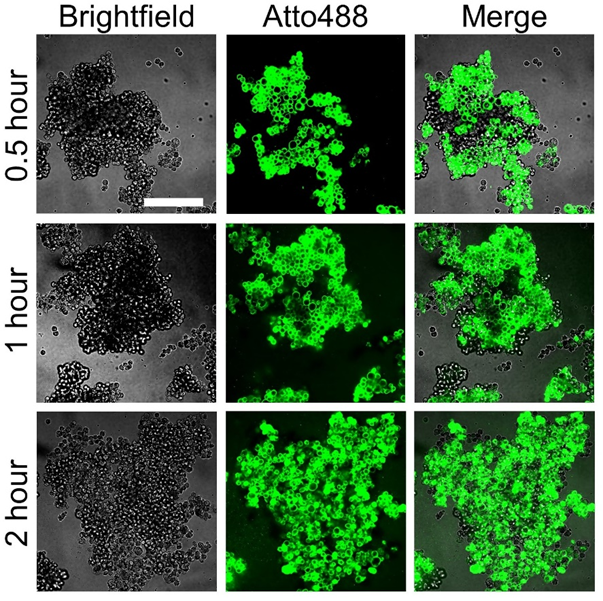
**

Supplementary Figure 3. Representative confocal fluorescence images after incubating 2.0 µM of Atto488-labeled lipid-DNA conjugates with MCF-7 spheroids for 0.5–2.0 hours at room temperature. Scale bar, 200 µm.


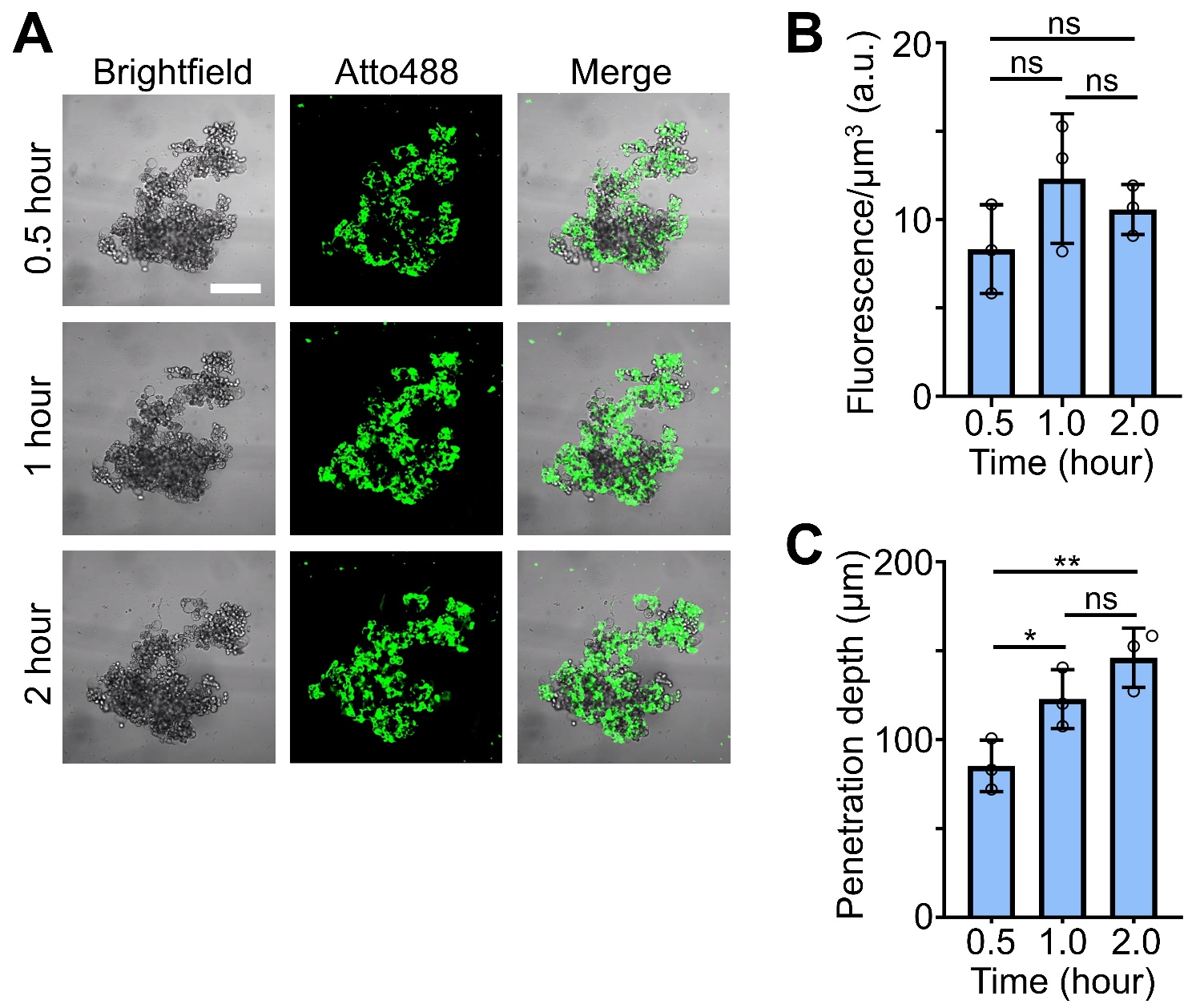


Supplementary Figure 4. (A) Representative confocal fluorescence images after incubating 2.0 µM of Atto488-labeled lipid-DNA conjugates for 0.5–2.0 hours at room temperature with 5-day-cultured MCF-7 spheroids in a 96-well ultra-low attachment U-bottom cell culture plate. Scale bar, 200 µm. (B) The corresponding average fluorescence intensities of the lipid-DNA modification region and (C) the penetration depth of lipid-DNA after incubating 2.0 µM of Atto488-labeled lipid-DNA conjugates for 0.5–2.0 hours at room temperature with 5-day-cultured MCF-7 spheroids. The low average fluorescence intensities are likely due to the use of U-bottom plate for the direct imaging, which is not an optimal plate for confocal. Shown are the mean and SD values from images taken from three spheroids in each case. Two-tailed student’s t-test: **, p<0.01; *, p<0.05. ns, not significant, p>0.05.


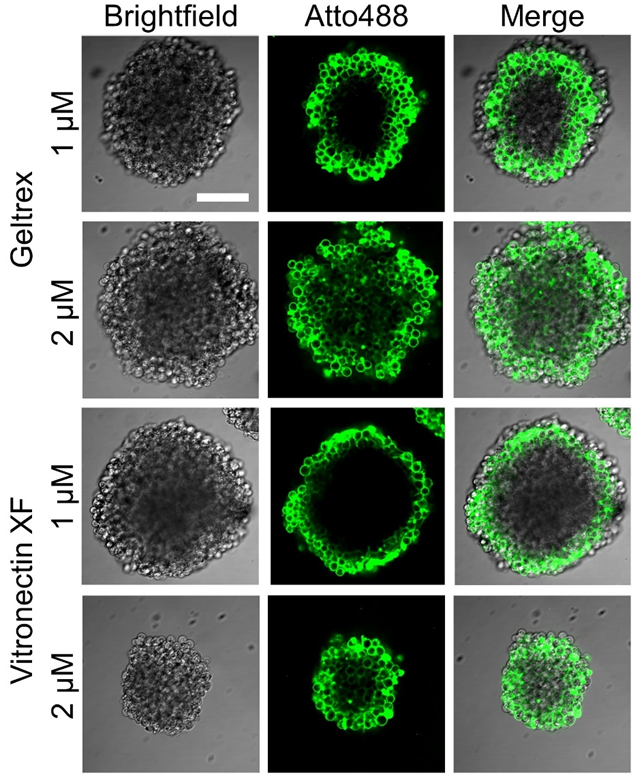


Supplementary Figure 5. Representative confocal fluorescence images after incubating 1.0 µM or 2.0 µM of Atto488-labeled lipid-DNA conjugates with Geltrex^TM^- or vitronectin XF^TM^-based H9 hESC EBs for 90 minutes at room temperature. Scale bar: 100 µm.


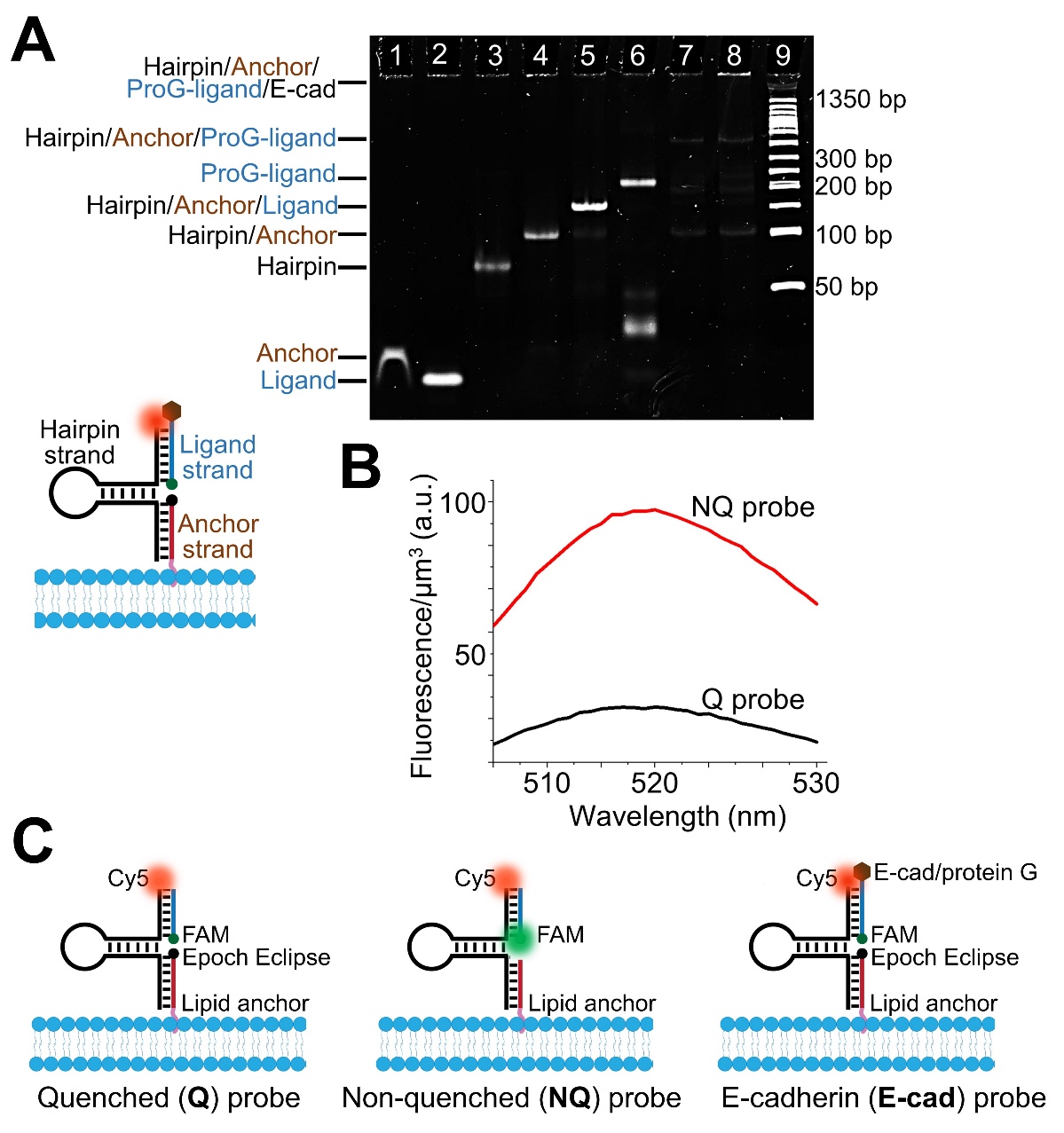


Supplementary Figure 6. (A) 10% native polyacrylamide gel electrophoresis analysis of DNA-based molecular tension probes. Lane 1, anchor strand; Lane 2, ligand strand; Lane 3, hairpin strand; Lane 4, anchor + hairpin strand; Lane 5, anchor + hairpin + ligand strand; Lane 6: ProG-modified ligand strand (the lower band may be due to the dimerization of ligand strands from disulfide bond formation); Lane 7: anchor + hairpin + ProG-modified ligand strand; Lane 8, anchor + hairpin + ProG-modified-ligand strand + E-cadherin; Lane 9: 50 bp DNA ladder. 10 µL of 1 µM DNA were loaded in each lane of Lane 2–8. 5 µL of 10 µg/mL DNA ladder was loaded in Lane 9. (B) Fluorescence emission spectra of 1 µM of NQ (quencher-free) and Q probes after excitation at 488 nm. (C) Illustration of the secondary structures of the quenched Q probe, non-quenched NQ probe, and E-cadherin E-cad probe. The E-cad probe is used to measure E-cadherin-mediated intercellular tensile forces. The NQ and Q probes contain the same DNA sequence and force tolerance range as that of the E-cad probe. However, these NQ and Q probes were not modified with the protein G or recombinant E-cadherin ligand, and thus not measuring E-cadherin-mediated forces. The NQ (without quencher) and Q probes were designed to indicate the maximum and minimum ratiometric signals, respectively, acting as the positive and negative controls.


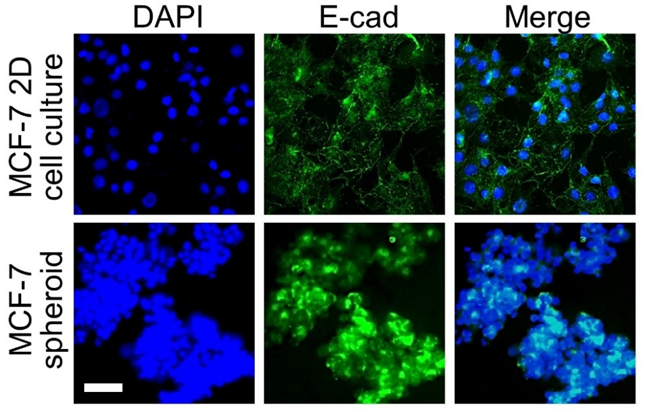


Supplementary Figure 7. After fixing MCF-7 cell culture or MCF-7 spheroids, representative immunofluorescence staining images are shown after an overnight 4°C incubation with primary E-cadherin antibody and then another 1-hour incubation with Atto488-labelled secondary antibody at room temperature. Scale bar, 50 µm.


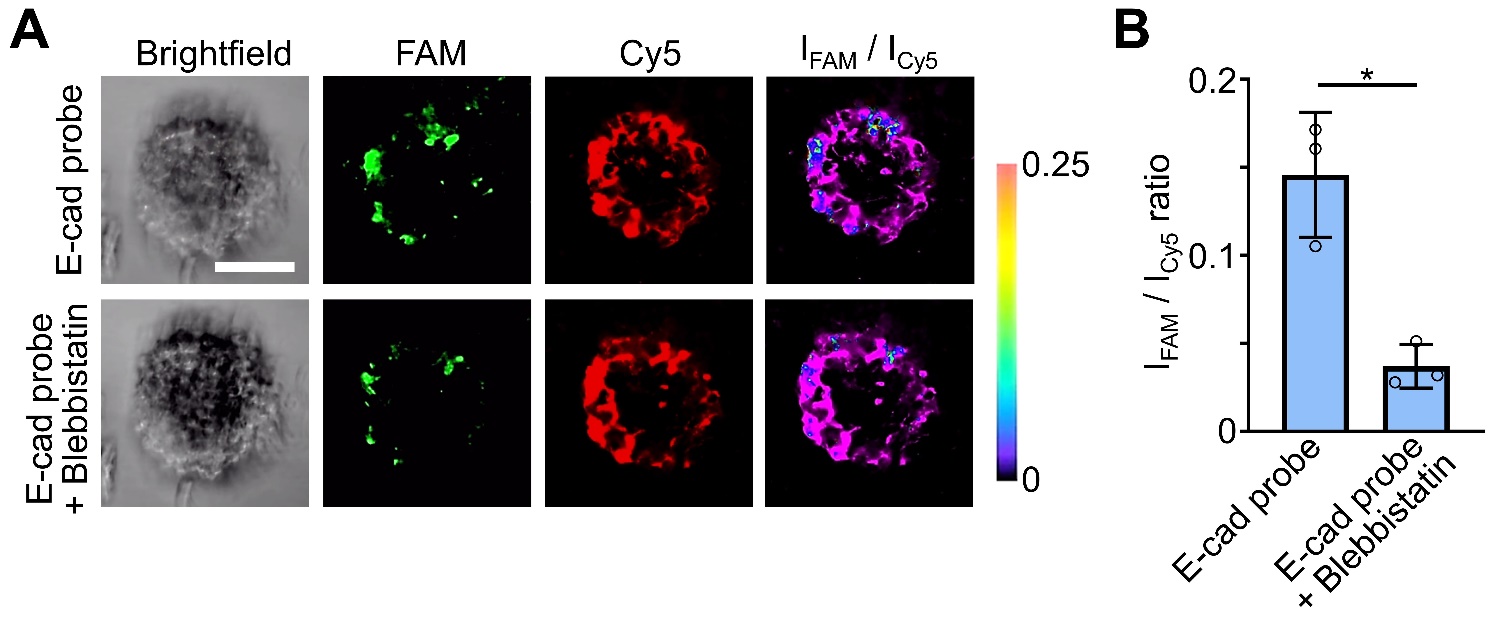


**Supplementary Figure 8.**  (**A**) Representative confocal fluorescence images and pseudo-colored FAM/Cy5 ratiometric images before and after the treatment of vitronectin XF^TM^-based H9 hESC EBs with 25 µM of blebbistatin for 10 minutes. 0.5 µM of E-cad probe was incubated with these EB samples for 30 minutes at room temperature before the addition of blebbistatin. Scale bar: 100 µm. **(B)** The average FAM/Cy5 ratiometric fluorescence intensities within H9 hESC EBs after incubation with 0.5 µM of E-cad probe for 30 minutes at room temperature and that after 10 minutes of blebbistatin treatment. Shown are the mean and SD values from images taken from three EBs in each case. Two-tailed student’s t-test: *, *p*<0.05.

# Supplementary Video

Z-stacking video of the embryoid body shown in Figure S5 after incubating 2 µM of Atto488-labeled lipid-DNA conjugates with vitronectin XF^TM^-based H9 hESC EBs for 90 minutes at room temperature.
